# 

**Published:** 1959-01

**Authors:** 


### Contents

| | | Page |
|---|---|---|
| THE ELIMINATION OF PILTDOWN MAN | *Mr. Hanbury Hazell* | 1 |
| RECENT DEVELOPMENTS IN CARDIAC SURGERY | *Mr. R. E. Horton and Dr. J. Clutton-Brock* | 6 |
| RECENT WORK ON THE DEMYELINATING DISEASES | *Dr. N. S. Alcock* | 17 |
| PROLONGED COMA IN BARBITURATE POISONING | *Dr. S. G. Flavell Matts and Dr. H. G. Handel* | 20 |
| HERPES ZOSTER WITH CHICKEN-POX AND LYMPHOSARCOMA | *Clinical Pathological Conference* | 22 |
| NOTES AND NEWS | . . . . . . . | 27 |
| APPOINTMENTS | . . . . . . . . | 27 |

